# Differential Effects of Mutations on the Transport Properties of the Na^+^/H^+^ Antiporter NhaA from Escherichia coli*

**DOI:** 10.1074/jbc.M113.484071

**Published:** 2013-07-08

**Authors:** Thomas Mager, Markus Braner, Bastian Kubsch, Lina Hatahet, Dudu Alkoby, Abraham Rimon, Etana Padan, Klaus Fendler

**Affiliations:** From the ‡ Max-Planck-Institut für Biophysik, 60438 Frankfurt/Main, Germany and; the § Institute of Life Sciences, Hebrew University of Jerusalem, 91904 Jerusalem, Israel

**Keywords:** Electrophysiology, Membrane Transport, pH Regulation, Site-directed Mutagenesis, Sodium-Proton Exchange, Transport Mechanism

## Abstract

Na^+^/H^+^ antiporters show a marked pH dependence, which is important for their physiological function in eukaryotic and prokaryotic cells. In NhaA, the *Escherichia coli* Na^+^/H^+^ antiporter, specific single site mutations modulating the pH profile of the transporter have been described in the past. To clarify the mechanism by which these mutations influence the pH dependence of NhaA, the substrate dependence of the kinetics of selected NhaA variants was electrophysiologically investigated and analyzed with a kinetic model. It is shown that the mutations affect NhaA activity in quite different ways by changing the properties of the binding site or the dynamics of the transporter. In the first case, p*K* and/or *K*_*D*_^Na^ are altered, and in the second case, the rate constants of the conformational transition between the inside and the outside open conformation are modified. It is shown that residues as far apart as 15–20 Å from the binding site can have a significant impact on the dynamics of the conformational transitions or on the binding properties of NhaA. The implications of these results for the pH regulation mechanism of NhaA are discussed.

## Teaser

**Background:** NhaA of *Escherichia coli* can serve as an example for pH regulation and transport mechanism in Na^+^/H^+^ exchangers.

**Results:** Different mutations alter Na^+^ and/or H^+^ binding or conformational dynamics.

**Conclusion:** Mutations in different segments of the protein change its pH profile by affecting different aspects of its transport mechanism.

**Significance:** Single site mutations and kinetic analysis reveal determinants of Na^+^/H^+^ exchanger mechanism.

## Introduction

Na^+^/H^+^ antiporters are crucial for survival in all biological kingdoms, as they control the intracellular sodium and proton concentration (1–3). For more than 20 years, NhaA from *Escherichia coli* served as a prototype for Na^+^/H^+^ antiporters. Moreover, NhaA is an example for the general mechanistic properties of transporters. The transport activity of NhaA shows a pronounced pH dependence (4, 5). On the basis of a large number of functional and structural investigations, a model of allosteric pH regulation was proposed for NhaA (6, 7). It was hypothesized that the pH-dependent change of the transport activity is due to the transition of the transporter from an inactive to an active conformation. Furthermore, the existence of a cytoplasmically located pH sensor was postulated. It is assumed that the pH sensor senses the intrabacterial pH and mediates the transition from an inactive conformation to an active conformation. Many pH-dependent conformational changes were revealed in various regions of the protein (8).

Recent electrophysiological analysis of NhaA in combination with kinetic analysis has provided further insights into the transport function of the antiporter (9). These measurements show that the decrease of the transport activity at acidic pH values is due to an increase of the *K*_*m*_^Na^ and not due to a decrease of the *V*_max_ (9) and explain this behavior with competitive binding of the substrates to a common binding site. A kinetic model (9) is proposed that explains the substrate dependence of the transport activity over a large concentration range. Furthermore, it is demonstrated that lowering the pH at the sodium release side to pH 5 does not inactivate NhaA in the forward mode nor in the reverse mode (9), thus providing strong evidence that NhaA is not adopting an inactive conformation even at pH 5 at the cytoplasmic or periplasmic side.

That the competition mechanism is a realistic hypothesis is supported by the observation that some of the conformational transitions are not only pH- but also Na^+^-dependent (9). However, a site-directed fluorescence study of a single Trp NhaA (Trp-less F136W NhaA) has recently revealed a pH-induced conformational change that is independent of the ligand (10). The two mechanisms of pH regulation described above represent obviously different mechanistic concepts, although they are not mutually exclusive and the conformational transitions they rely on are not necessarily different.

A central argument for the existence of a pH sensor and the related allosteric model of pH regulation is based on the drastic effects that mutations in regions very remote from the cation-binding site (2, 8, 11) exert on the pH profile of NhaA. In this study, we therefore analyzed a number of different NhaA variants with mutations remote from and close to the cation-binding site. Using solid supported membrane (SSM)^2^-based electrophysiology combined with a kinetic model, a more detailed insight into the transporter function is possible than with previous techniques.

**FIGURE 1.**
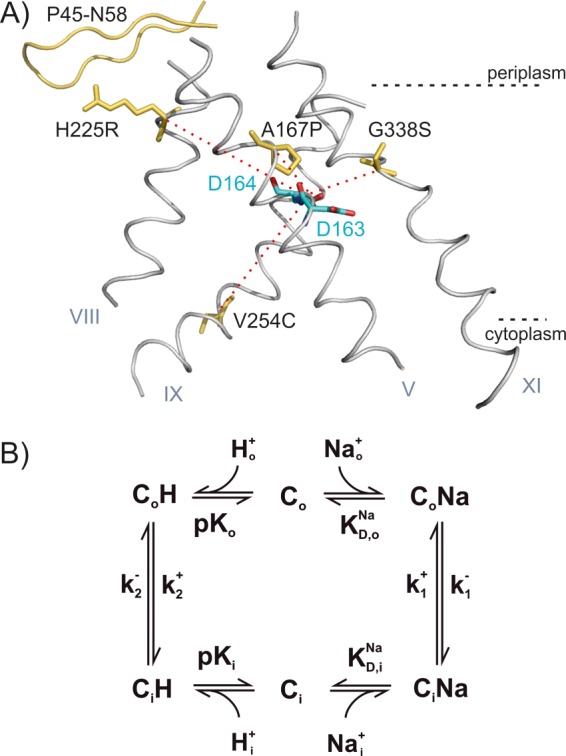
*A,* helices V, VIII, IX. and XI in the structure of NhaA with the modified amino acid residues indicated in *yellow*. The two aspartates of the putative cation-binding site (Asp-163 and Asp-164) are shown in *blue* and *red*. The β-sheet P45-N58 is drawn in *yellow*. The *red dotted lines* indicate the trajectories from the mutated residues to the cation-binding site. *B,* kinetic model for Na^+^/H^+^ antiport. In the “forward mode,” the outside directed transporter C_o_ binds H^+^ (this could be one or two H^+^ ions, and for simplicity only one is considered in the model) from the periplasm (H_o_), performs a conformational transition to the inward directed form C_i_, and releases H^+^ to the cytoplasm. Subsequently, Na^+^ is bound from the cytoplasm (Na_i_) and released to the periplasm.

To investigate the effect of mutations remote from the active site on the pH profile, we functionally characterized the mutants V254C NhaA and H225R NhaA (Fig. 1*A*). Val-254 is located at the cytoplasmic end of helix IX, which is part of the postulated pH sensor region. His-225 is in helix VIII, which is flanking the periplasmic funnel. These mutants showed an altered pH dependence at preceding biochemical investigations (7, 12–15).

NhaA is a dimer, and the dimerization state was suggested to be pH-dependent (16) and important for the stability of the antiporter under stress conditions (17). Therefore, we also investigated the effect of dimerization on the transport properties of NhaA by using the monomeric mutant ΔP45-N58 NhaA (18). Finally, we functionally characterized the mutant A167P. This mutation is located at transmembrane helix V in close proximity to the binding site (Fig. 1*A*). Surprisingly, this novel mutant did not show the characteristic property expected for an electrogenic transporter such as wild type (WT) NhaA with a stoichiometry of 2H^+^/1Na^+^. In particular, the enhancement of Na^+^ uptake rate by collapsing the membrane potential is drastically smaller in A167P than in WT NhaA. The following study explains the differential effects of mutations on the transport properties of NhaA from *E. coli* on the basis of a detailed kinetic analysis.

## EXPERIMENTAL PROCEDURES

### 

#### 

##### Overexpression, Purification, and Reconstitution of NhaA Variants

The mutants (Fig. 1*A*) ΔP45-N58 (18), V254C (13), Cys-less (19), and H225R (12) were previously isolated and characterized experimentally. Isolation of A167P and its characteristics will be described elsewhere.^3^ C-terminally His-tagged A167P, ΔP45-N58, V254C, Cys-less, and H225R NhaA were overexpressed and purified on a Ni^2+^-nitrilotriacetic acid column as described previously (20). Purified protein in 25 mm potassium acetate (pH 5), 100 mm KCl, 5 mm MgCl_2_, 0.03% dodecyl β-d-maltoside was stored at 4 °C until reconstitution. Reconstitution of the variant protein was carried out with *E. coli* phospholipids (Avanti Polar Lipids) using polystyrene beads for detergent removal at a lipid to protein mass ratio of 10 (9). The final proteoliposomes were adjusted to a lipid concentration of 5 mg/ml, frozen, and stored at −80 °C until use. Prior to the SSM measurements, the samples were gently sonicated with cooling breaks on ice.

##### Production of ΔP45-N58 and V254C NhaA Inside-out Vesicles

ISO membrane vesicles were produced as described (9) by disruption of V254C NhaA or of ΔP45-N58 NhaA overexpressing cells with a French press (20.000 p.s.i.). The ISO membrane vesicles were further purified by metal affinity two-phase partitioning according to Ref. 21.

##### Reconstitution of NhaA into Proteoliposomes and Measurement of ΔpH-driven ^22^Na Uptake

NhaA proteoliposomes were reconstituted, and ΔpH-driven ^22^Na uptake was determined as described previously (4). All experiments were repeated at least twice with practically identical results.

##### SSM-based Electrophysiology

SSM measurements were performed as described previously (22). Briefly, 45 μl of proteoliposomes at a lipid concentration of 5 mg/ml or ISO membrane vesicles at a total membrane protein concentration of 1.5–3 mg/ml were adsorbed to an octadecanethiol/phospholipid hybrid bilayer on a gold surface (sensor). Proteoliposomes were allowed to adsorb to the sensor for 2–3 h. Adsorption of the ISO membrane vesicles was carried out for 3–12 h. Electrogenic transport was initiated by a rapid concentration jump of Na^+^ ions (sodium gradient-driven transport) using a single solution exchange protocol (nonactivating solution, 0.5 s; activating solution, 0.5 s; nonactivating solution, 0.5 s). Currents recorded throughout the measurements were amplified with a current amplifier set to a gain of 10^8^–10^10^ V/A and low pass filtering at 2000 Hz. To minimize Na^+^ concentration jump artifacts, sodium was compensated for by an equal amount on an inert cation (K^+^) in the nonactivating solution. For the same reason, the ionic strength was kept high by adding K^+^ to a total cation concentration of 305 mm. Details concerning the composition of the solutions are given in the figure legends.

##### Data Analysis

Na^+^ concentration jumps on the SSM are accompanied by transient currents of positive signs (solution exchange artifact). In contrast, NhaA transporter currents after a Na^+^ concentration jump have a negative sign. The negative transient currents were subtracted by the positive solution exchange artifacts to get transient currents that reflect only the transport activity (9). The Na^+^ dependences of the peak currents (three data sets) were normalized to the corresponding *V*_max_ values resulting from a hyperbolic fit to the individual data set and then averaged. To describe the pH dependence of the transient currents, peak currents *I*_peak_ were recorded at 10 and 100 mm Na^+^. Normalization of the data was performed according to Ref. 9.

For the fits with the symmetric kinetic model the parameters were set to those shown in Model 1,

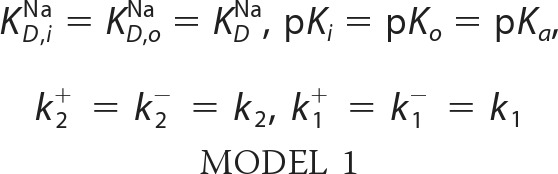
 For the fits with the asymmetric kinetic model the parameters were set those shown in Model 2,

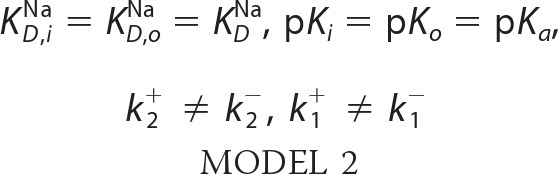
 For a detailed description of the kinetic model see Ref. 9.

## RESULTS

**FIGURE 2.**
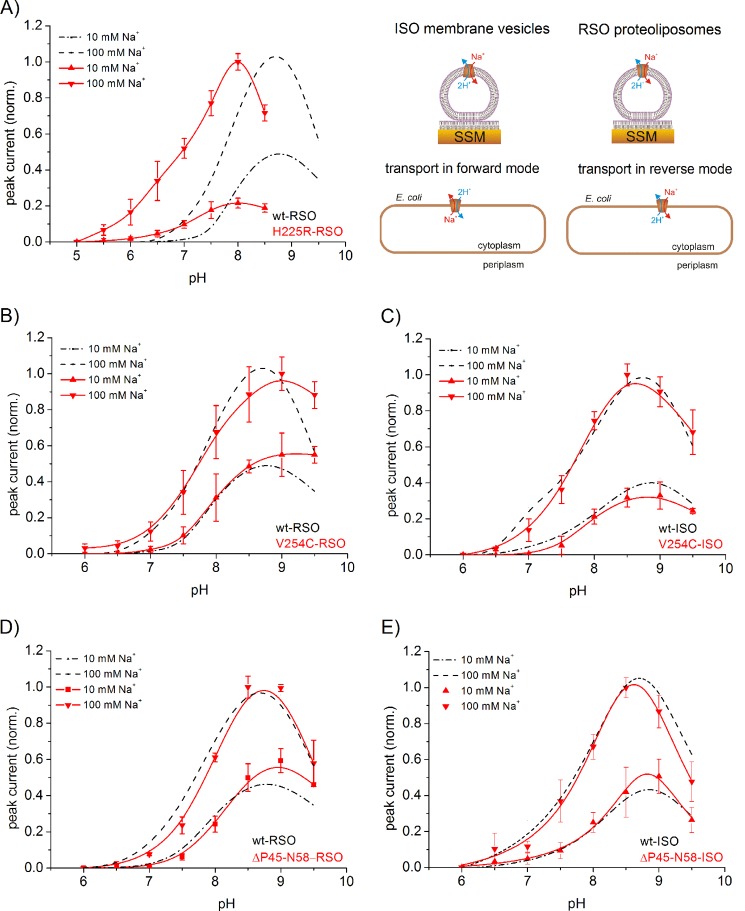
**pH dependences of transient currents obtained with H225R NhaA, V254C NhaA, and ΔP45-N58 NhaA after a Na^+^ concentration jump.** Shown are the normalized peak currents at indicated pH values after a 10 or 100 mm Na^+^ concentration jump. As a guide to the eye, the pH dependences were fitted with a Voigt function. Data obtained with RSO proteoliposomes of H225R NhaA (*A*), V254C NhaA (*B*), and ΔP45-N58 NhaA (*D*) as well as data obtained with ISO membrane vesicles of V254C NhaA (*C*) and ΔP45-N58 NhaA (*E*) are shown in *red*. For comparison, the WT NhaA pH dependences are included in *black* (taken from Ref. 9). In the *upper right panel,* a graphic representation of the transport modes of NhaA in the bacterial cell and under experimental conditions is shown. The *graphs* show average values from recordings using three individual sensors and the corresponding S.D. Currents were normalized as described under “Experimental Procedures.” For the Na^+^ concentration jumps, activating solutions containing 10 mm NaCl and 290 mm KCl or 100 mm NaCl and 200 mm KCl titrated to the indicated pH values with HCl or Tris were used. The nonactivating solutions contained 300 mm KCl instead. In addition, all buffers contained 5 mm MgCl_2_, 25 mm Tris, 25 mm MOPS, 25 mm MES, and 1 mm DTT.

To perform a kinetic analysis of the transporter activity of ΔP45-N58, V254C, H225R, and A167P NhaA, right-side out (RSO) proteoliposomes, exposing the periplasmic side of the transporter to the outside, were prepared and investigated using SSM-based electrophysiology. Note that NhaA spontaneously adopts an RSO orientation in proteoliposomes (23). V254C NhaA and ΔP45-N58 NhaA were also studied in the opposite transport direction using ISO membrane vesicles derived from bacteria overexpressing the corresponding mutant proteins and exposing the cytoplasmic side to the outside (Fig. 2*A, right panel*) (9). Membrane vesicles prepared by French press cell disruption have a preferential ISO orientation (24). A complete ISO orientation of the vesicles was further ensured using an extraction procedure with nickel-nitrilotriacetic acid-tagged beads (21). Transport was initiated by a rapid Na^+^ concentration jump. The ensuing transporter activity generates a transient current. Its peak value is a reliable estimate of the steady-state transport activity of NhaA (23). By applying an inwardly directed Na^+^ gradient to the RSO proteoliposomes, transport against physiological transport direction (reverse mode) was investigated (Fig. 2, *A, B,* and *D*). Transport in physiological transport direction (forward mode) was investigated by applying an inwardly directed Na^+^ gradient (Na^+^ inside = 0) to ISO membrane vesicles (Fig. 2, *C* and *E*).

### 

#### 

##### pH Dependence of H225R, ΔP45-N58, and V254C NhaA

Peak currents corresponding to the reverse mode were recorded after 10 or 100 mm Na^+^ concentration jumps applied to RSO proteoliposomes at pH values from 6 to 9.5 for ΔP45-N58 (Fig. 2*D*) and V254C NhaA (Fig. 2*B*) and at pH values from 5 to 9.5 for H225R NhaA (Fig. 2*A*). No pre-steady-state current components were observed throughout the displayed pH range. V254C NhaA and ΔP45-N58 NhaA were also investigated in the forward mode (Fig. 2, *C* and *E*) using ISO membrane vesicles.

The Na^+^ gradient-driven transport resulted in negative transient currents in agreement with the 2H^+^/1Na^+^ stoichiometry of NhaA (5). The peak currents at optimal pH and 100 mm Na^+^ were −1.0 ± 0.5 nA for H225R NhaA (pH 8), −3.5 ± 1.0 nA for ΔP45-N58 NhaA (pH 9), and −6 ± 3 nA for V254C NhaA (pH 9). The relatively large variation of the absolute current values stems from the fact that the amount of adsorbed proteoliposomes varies between different sensors.

The pH dependences of ΔP45-N58 NhaA and WT NhaA in both transport directions were virtually the same (Fig. 2, *D* and *E*). However, the other mutants show clear alterations of the transport properties compared with WT NhaA (Fig. 2). Values for the pH of optimal transport, pH_opt_, are compared in Table 1. The pH dependence of H225R NhaA in the reverse mode was shifted by ∼1 pH unit to the acidic range compared with the wild type. In addition, the ratio of the peak currents after performing a 10 mm Na^+^ concentration jump and after a 100 mm Na^+^ concentration jump differed significantly from WT NhaA (Fig. 2*A*). For V254C NhaA, an alkaline shift to pH 9.0 was observed in the reverse mode (Fig. 2*B*). Moreover, in this mutant, the bell-shaped pH dependence was widened in comparison with WT. In contrast, in the forward mode the pH dependence of V254C NhaA and WT NhaA was virtually the same (Fig. 2*C*) demonstrating a clear asymmetric behavior of V254C NhaA. The mutation V254C was introduced in an NhaA variant with all cysteines being mutated to serines (Cys-less NhaA). Cys-less NhaA has the same transport properties like WT NhaA (19). To verify this by SSM measurements, the pH dependence of ΔNa-driven transport using reconstituted Cys-less NhaA was investigated between pH 6 and pH 9.5. In accordance with previous measurements, the pH dependences of WT NhaA and Cys-less NhaA were, in consideration of the experimental error, identical (data not shown).

**TABLE 1:** **Kinetic parameters of ΔNa^+^-driven transport using ISO and RSO preparations** Table lists the kinetic parameters of V254C NhaA, A167P NhaA, H225R NhaA, and ΔP45-N58 NhaA. The *K*_*m*_^Na^ values were determined at indicated pH values by the hyperbolic fit to the Na^+^ dependencies. For the Na^+^ concentration, jumps activating solutions containing *x* mm NaCl and (300 − *x*) mm KCl titrated to the indicated pH values with Tris were used. The nonactivating solutions contained 300 mm KCl instead. In addition, all buffers contained 5 mm MgCl_2_, 25 mm Tris, 25 mm MOPS, 25 mm MES, and 1 mm DTT. For comparison, *V*_max_, the average of the saturation values of the hyperbolic fit is shown. The pH of maximal peak current pH_opt_ was taken from the Voigt fit to the pH dependencies after a 100 mm Na^+^ concentration jump (Figs. 2 and 3*C*).

NhaA	Transport modus	pH	*K*_*m*_^Na^	pH_opt_	*V*_max_
WT*^a^*	Forward	8.5	14.5 ± 1.8 mm	8.7	
		7.5	99.3 ± 17.9 mm		
	Reverse	9	7.3 ± 1.1 mm	8.7	−8.0 ± 6.0 nA (pH 9)
		7.5	102.2 ± 6.5 mm		
V254C	Forward	8.5	15.9 ± 2.1 mm	8.7	
		7.5	91.4 ± 9.0 mm		
	Reverse	9	3.6 ± 0.9 mm	9.0	−3.3 ± 1.5 nA (pH 9)
		7.5	65.2 ± 18.4 mm		
ΔP45-N58	Forward	8.5	12.5 ± 2.3 mm	8.6	
		7.5	94.6 ± 38.9 mm		
	Reverse	9	5.3 ± 1.0 mm	8.7	−3.5 ± 1.0 nA (pH 9)
		7.5	62.8 ± 22.7 mm		
H225R	Reverse	8	106.6 ± 23.4 mm	7.9	−3.2 ± 1.8 nA (pH 8)
		7	154.5 ± 15.1 mm		
A167P	Reverse	8.5	45.8 ± 6.3 mm	8.6	−0.5 ± 0.3 nA (pH 8.5)
		7.5	285.4 ± 136.0 mm		

*^a^* Values for WT NhaA were taken from Ref. 9.

##### Na^+^ Dependence of H225R, ΔP45-N58, and V254C NhaA at Optimal and Low pH

For the determination of *K*_*m*_^Na^, averaged data of different sensors were used. The Na^+^ dependences of Na^+^ gradient-driven reverse mode transport of the mutants were determined at pH values, where the transport activity is maximal, and at more acidic pH values, where the transport activity is reduced (Fig. 2). In addition, forward mode transport of V254C NhaA and ΔP45-N58 NhaA was investigated for comparison. The individual data sets obtained with different sensors were fitted with a hyperbolic model function with a half-saturation concentration *K*_*m*_^Na^ and a saturation value *V*_max_. As discussed above, comparison of *V*_max_ is associated with large errors (see Table 1) because different sensors were used in each experiment and different amounts of proteoliposomes were absorbed per sensor. Nevertheless, the values demonstrate that mutants and WT have approximately the same transport capacity in the reverse mode. A comparison with forward mode is also not possible, because the concentration of NhaA in the ISO membrane vesicles was not determined. However, a direct comparison of *V*_max_ at different pH values is possible, by comparing data determined with the same sensor. Performing this analysis led to the conclusion that in both transport directions, *V*_max_ did not change significantly with pH (data not shown), but *K*_*m*_^Na^ did. This finding has already proven to be valid for WT NhaA and G338S NhaA and is a strong indicator for Na^+^/H^+^ competition being a significant reason for the decrease of transport activity at low pH (9).

##### Transport Properties of A167P NhaA

**FIGURE 3.**
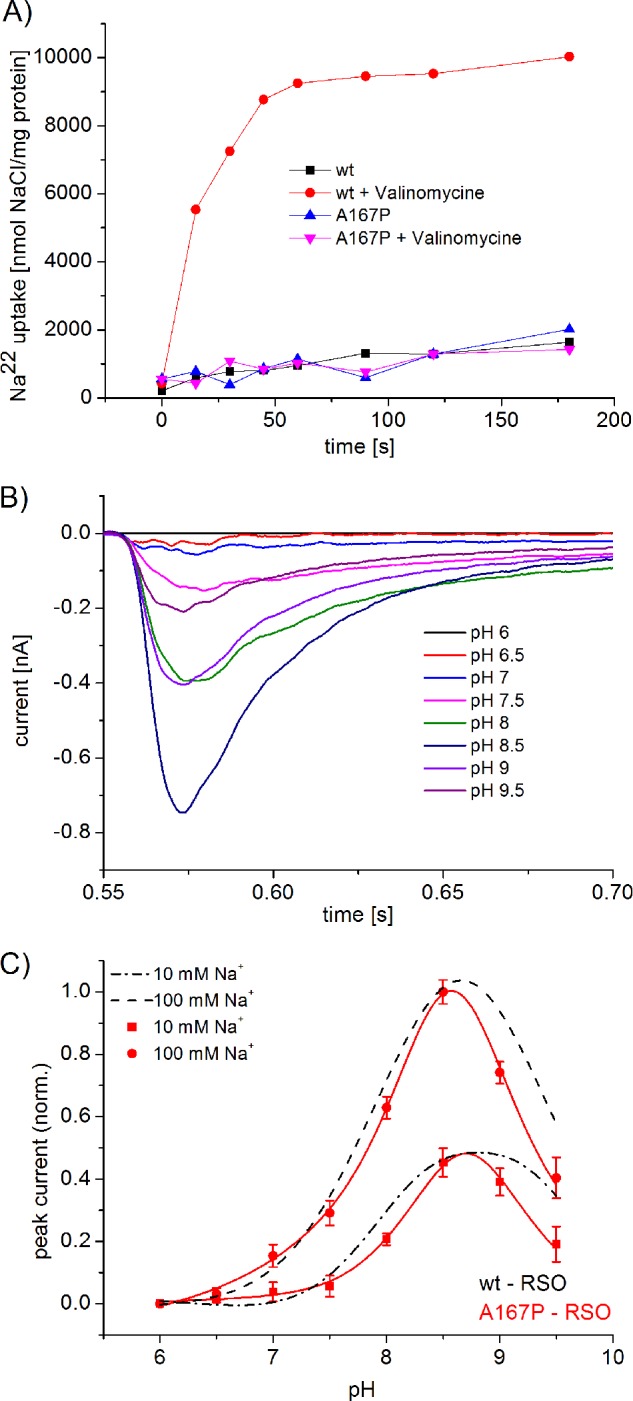
*A,* ^22^Na uptake of A167P NhaA reconstituted in liposomes in comparison with WT NhaA with and without (+*valinomycin*), a membrane potential. NhaA proteoliposomes were reconstituted, and ΔpH-driven ^22^Na uptake was determined as described previously (4). All experiments were repeated at least twice with practically identical results. *B* and *C,* pH dependences of transient currents obtained with A167P proteoliposomes after a Na^+^ concentration jump. *B,* transient currents after 100 mm Na^+^ concentration jump at indicated pH values. The transient currents were artifact corrected as described under “Experimental Procedures.” *C,* normalized peak currents obtained with A167P proteoliposomes (in *red*) at indicated pH values after a 10 mm or 100 mm Na^+^ concentration jump. For comparison, the normalized peak currents obtained with WT NhaA proteoliposomes are shown in *black*. The *graph* shows average values from recordings using three individual sensors and the corresponding S.D. Currents were normalized as described under “Experimental Procedures.” For the Na^+^ concentration jumps, activating solutions containing 10 mm NaCl and 290 mm KCl or 100 mm NaCl and 200 mm KCl titrated to the indicated pH values when HCl or Tris were used. The nonactivating solutions contained 300 mm KCl instead. In addition, all buffers contained 5 mm MgCl_2_, 25 mm Tris, 25 mm MOPS, 25 mm MES, and 1 mm DTT.

As expected for an electrogenic transporter with a stoichiometry of 2H^+^/1Na^+^, WT NhaA reconstituted in proteoliposomes shows a dramatic increase of the ΔpH-driven Na^22^ uptake rate upon addition of a permeant ion (K^+^ in the presence of valinomycin) to collapse the membrane potential (Fig. 3*A*). In marked contrast the mutant A167P, in a similar experiment, was nearly indifferent to a change in the membrane potential. This difference can suggest that variant A167P is less electrogenic as compared with the WT or is an electroneutral antiporter. Surprisingly, transporter currents were measured with A167P proteoliposomes using SSM-based electrophysiology (Fig. 3*B*). Hence, A167P NhaA is electrogenic, ruling out that it is an electroneutral transporter. The pH dependence of the mutant is not shifted in comparison with the wild type (Fig. 3*C*). However, A167P NhaA differs from the WT transporter in two important aspects. 1) It has a strongly reduced transport capacity compared with WT, which is apparent in the Na^+^ uptake as well as the electrical measurements (Fig. 3*A* and Table 1). 2) It shows a stronger down-regulation in the alkaline range compared with WT NhaA (Fig. 3*C*).

##### Comparison of the Kinetic Parameters

The comparison in Table 1 reveals the phenomenological divergence of the mutant transport properties. Optimal transport properties were found for most mutants and the WT in a narrow pH range 8.6–9.0 and a *K*_*m*_^Na^ for Na^+^ ranging from 3.6 to 14.5. Exceptions are the acid-shifted H225R mutation and the low Na^+^ affinity mutations H225R and A167P. Although turnover *V*_max_ is associated with a large standard deviation, the general picture is that of only small changes afflicted to transporter turnover by the mutations except A167P, which has an ∼6-fold lower turnover than the other variants. All investigated variants showed a *K*_*m*_^Na^ increase in acidic environment albeit to different degrees indicative of H^+^/Na^+^ competition for the transporter-binding site. Phenomenological kinetic parameters as compiled in Table 1 are compound parameters affected by different mechanistic aspects of the transporter. A deeper insight into the transport mechanism may be obtained using a kinetic model-based analysis as will be discussed below.

## DISCUSSION

To test the molecular determinants of the pH regulation of the NhaA Na^+^/H^+^ exchanger, we have studied a number of mutants with an altered pH dependence using SSM-based electrophysiology. In this analysis, we also included the previously described G338S mutant (4, 9) to provide a more complete view on NhaA variants with a modified pH profile. We will discuss the obtained experimental results in the light of previously reported data and, in addition, analyze the data on the basis of a kinetic steady-state model that we have developed. The different mutations have a quite distinct effect on the transport properties of NhaA. They can affect the properties of the binding site and/or the dynamics of the transporter represented by the rate constants of the conformational transitions between the inside- and the outside-open state. The results have implications for the mechanism of pH regulation of NhaA.

Fig. 1 shows that the different mutations are localized in different parts of the transporter as follows: in helices V (A167P), VIII (H225R), IX (V254C), and XI (G338S). They range from being as close as ∼7 Å (G338S) from the putative substrate-binding site of NhaA to as far as ∼20 Å (V254C). Because NhaA is a dimer and mutant V254C is in the interface between the two monomers (25), we also studied a monomeric NhaA variant to test whether the transport properties of a mutant could be the result of an altered interaction of the NhaA monomers. The phenomenological parameters of the mutants summarized in Table 1 show a variety of differences in transport properties but also common features compared with the WT. A common theme in WT and mutants is an increased *K*_*m*_^Na^ at acidic pH, although the effect in H225R is rather small. Distinct differences between the investigated transporters are also apparent. For example, V254C NhaA has a significant functional asymmetry with respect to transport direction as demonstrated in Fig. 2, *B* and *C*. We find no acidic shift of the pH profile as reported previously (13) but rather a moderate alkaline shift in the reverse transport direction (Table 1). In contrast, H225R NhaA is characterized by a prominent *K*_*m*_^Na^ increase for both substrates, Na^+^ and H^+^ (see the pH_opt_ decrease in Table 1). A *K*_*m*_^Na^ increase but somewhat less pronounced is found for A167P. In addition, this variant has a slower turnover as demonstrated by a six times lower *V*_max_ compared with the other variants. The variety of effects of mutations in the different positions already shows that not only the binding site is affected but also dynamic effects have to be considered. Therefore, a more profound analysis was attempted using a kinetic model from which a more decisive picture emerges.

### 

#### 

##### Kinetic Model

We have previously proposed a kinetic model (Fig. 1*B*), which was successfully applied for the description of the transport properties of the NhaA Na^+^/H^+^ exchanger, as measured electrophysiologically (9). This model consists of only a single common binding site for Na^+^ and H^+^ alternating across the membrane (26, 27). Substrate binding and release is described by dissociation constants for Na^+^ (*K*_*D*_^Na^) and H^+^ (p*K_a_*), because the substrates are assumed to be in rapid equilibrium. The rate-determining reactions are the conformational transitions between the outward facing conformation (C_o_) and the inward facing conformation (C_i_) described by their forward and backward rate constants in the sodium-bound form (*k*_1_^+^, *k*_1_^−^) and in the proton bound form (*k*_2_^+^, *k*_2_^−^). For WT NhaA, symmetric transport properties were experimentally found. Consequently, a symmetric kinetic model was postulated with symmetric forward and backward rate constants as well as symmetric outside and inside substrate dissociation constants (9). Indeed, it was possible to describe the Na^+^ and H^+^ dependence of WT NhaA in the whole concentration range with the kinetic model without an additional allosteric pH regulation mechanism (9).

**FIGURE 4.**
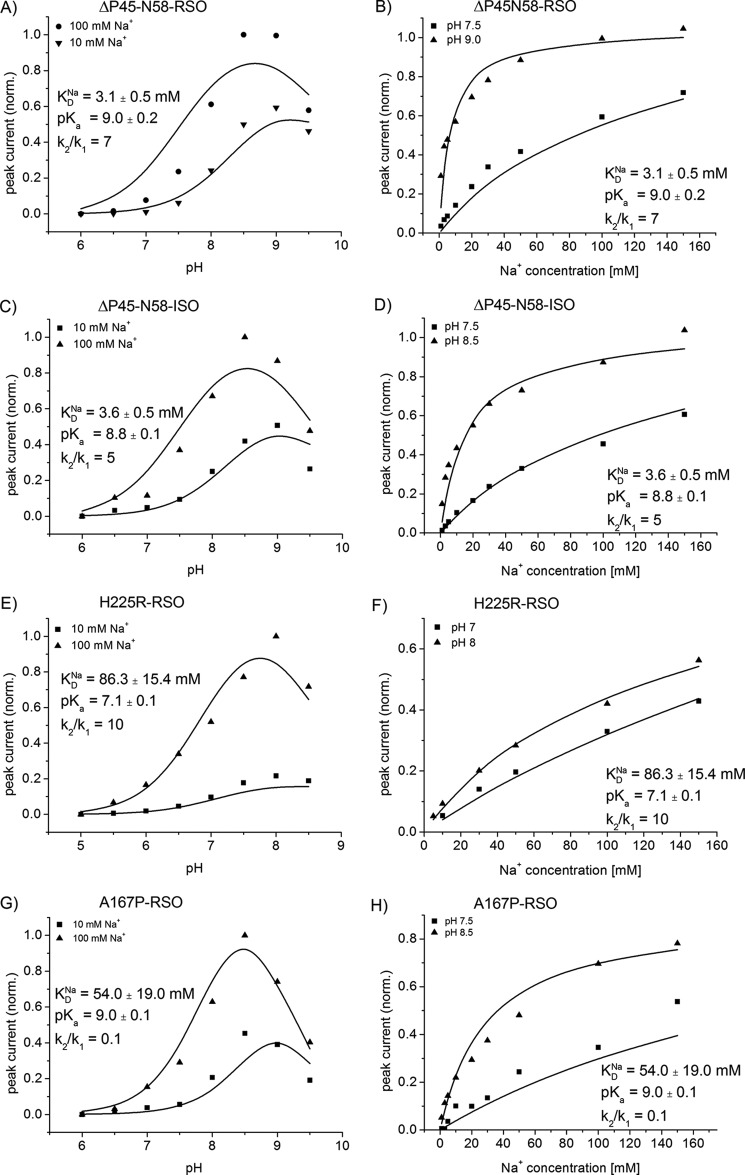
**Simultaneous fit of the pH dependences at indicated sodium concentrations and the sodium dependences at indicated pH values to the kinetic model.** The *graphs* show sodium jump-induced peak currents using ΔP45-N58 NhaA RSO proteoliposomes (*A* and *B*), ΔP45-N58 NhaA ISO membrane vesicles (*C* and *D*), H225R NhaA RSO proteoliposomes (*E* and *F*), and A167P RSO proteoliposomes (*G* and *H*). Data and conditions are as in Fig. 2 (pH dependences) and Table 1 (Na^+^ dependences). The *solid line* is a fit to the kinetic model described in the text. The kinetic parameters determined by the fit are shown in the figure.

**FIGURE 5.**
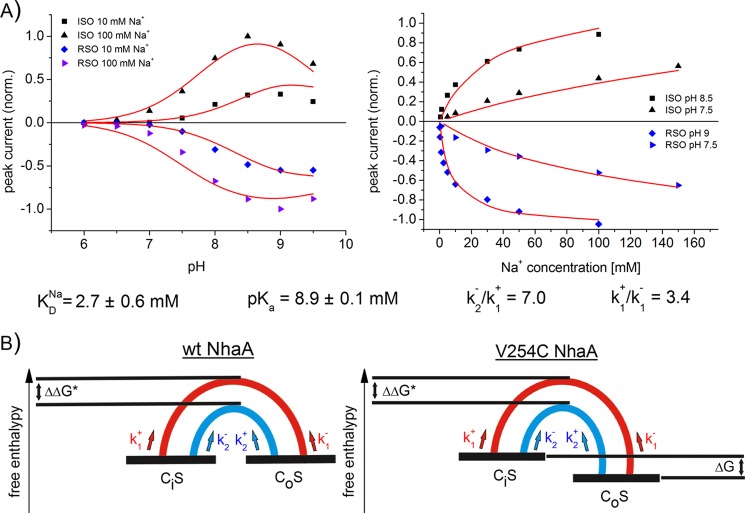
*A,* fit of the sodium jump-induced peak currents of V254C NhaA to the asymmetric kinetic model. Simultaneous fit of the pH dependences was at indicated sodium concentrations, and the sodium dependences was at indicated pH values of V254C NhaA in both transport directions. The *solid line* is a fit to the asymmetric kinetic model described in the text. The kinetic parameters obtained by the fit are as follows: *K*_*D*_^Na^ = 2.7 ± 0.6 mm, p*K_a_* = 8.9 ± 0.1 mm, *k*_2_^−^/*k*_1_^+^ = 7.0, and *k*_1_^+^/*k*_1_^−^ = 3.4. Data and conditions as in Fig. 2 and Table 1. *B,* energy diagram of WT NhaA and V254C NhaA in a simplified representation. CS is the transporter in the substrate-bound form, S = Na^+^ (*red*) or S = H^+^ (*blue*). The subscript *i* (inside) marks the state of the transporter opened to the cytoplasm, and the subscript *o* (outside) marks the state of the transporter opened to the periplasm. The half-cycles are graphical representations of the energy barriers between the inside opened and the outside opened conformation of the transporter in the sodium-bound form (*red*) or in the proton-bound form (*blue*). ΔΔG* is the difference of the activation enthalpies of the transitions states. Δ*G* is the free enthalpy difference of the inside opened and the outside opened conformation.

The fitted parameters determined for the different mutants and the WT are given in Figs. 4 and 5 and are summarized for comparison in Table 2. We observe three different phenotypes as follows: p*K*-shifted variants G338S and H225R, low Na^+^ affinity variants H225R and A167P, but also variants with modified conformation dynamics A167P and V254C. The latter is important because it demonstrates that a simple kinetic characterization based on *K*_*m*_^Na^ and *V*_max_ values can be misleading.

**TABLE 2:** **Parameters determined using the kinetic model of Fig. 1*B*** The kinetic parameters of H225R, A167P, and ΔP45-N58 NhaA were determined by a simultaneous fit of the pH and Na^+^ dependencies using the symmetric kinetic model (Fig. 4). For V254C NhaA, an asymmetric (asym.) kinetic model was used (Fig. 5). The corresponding kinetic parameters for WT NhaA and G338S NhaA were taken from Ref. 9. When forward and reverse modes were separately analyzed as in the case of WT, G338S, and ΔP45-N58, averaged values are given. Distances of the mutated amino acid to the binding site are given as distances from the Cα carbon of the amino acid to the C_γ_ carbon of Asp-164. Boldface values indicate parameters deviating significantly from the WT.

	Helix	*d*	*K*_*D*_^Na^	p*K_a_*	*k*_2_*/k*_1_
		Å			
WT			3.4	8.8	7
ΔP45-N58			3.3	8.9	6
G338S	XI	7.3	11	**7.0**	4
H225R	VIII	15.5	**86**	**7.1**	10
A167P	V	7.5	**54**	9.0	**0.1**
V254C	IX	20.4	2.7	8.9	**Asym.**

##### Kinetics of Monomeric NhaA Is Similar to the WT Dimer

In the mutant ΔP45-N58 NhaA, the antiparallel β-sheet, which is part of the dimer interface, is deleted (18, 28). Therefore, ΔP45-N58 NhaA adopts predominantly a monomeric form (18). We found that the pH dependences of ΔP45-N58 NhaA in both transport directions were virtually the same and similar to the WT (Fig. 2, *D* and *E*). Furthermore, in accordance with the literature, the monomeric mutant has the same transport properties like WT NhaA (18). Our results support the idea that the beneficial effect of dimerization under stress conditions is based on stability issues and not on a direct impact of dimerization on the functional properties of NhaA (17).

##### V245C NhaA, an Asymmetric Transporter

Mutation V254C is located at the cytoplasmic end of helix IX. This is part of the dimer interface (13, 28) and has been proposed to belong to the postulated pH sensor region (6). However, using SSM-based electrophysiology, V254C NhaA has the same pH dependence in the forward mode like WT-NhaA (Fig. 2*C*). Moreover, Na^+^/H^+^ competition seems to be responsible for acidic down-regulation as in the WT (Table 1). However, the pH profile is clearly different in the reverse mode, where very little alkaline down-regulation is observed (Fig. 2*B*).

Asymmetric behavior can be introduced into the kinetic model in two different ways as follows: via asymmetric substrate dissociation constants p*K_o_* ≠ p*K_i_* and/or *K*_*D, o*_^Na^ ≠ *K*_*D, i*_^Na^ or via asymmetric rate constants of the conformational transitions *k*_1_^+^ ≠ *k*_1_^−^ and/or *k*_2_^+^ ≠ *k*_2_^−^. Both approaches lead to kinetic models that reproduce the experimental data for V254C NhaA in forward and reverse mode equally well. Therefore, it is not possible *per se* to decide in favor of the two alternative models. In the following we will choose a kinetic model with orientation-independent substrate-binding constants p*K_o_* = p*K_i_* and/or *K*_*D, o*_^Na^ = *K*_*D, i*_^Na^, and we will assume that the asymmetry of the kinetic model is strictly brought about only by the rate constants. The rationale for this choice is discussed below.

Fig. 5*A* shows the simultaneous fit of the pH and sodium dependences of V254C NhaA in the forward and the reverse mode to the asymmetric kinetic model. It demonstrates that an appropriate description of the substrate dependences by the asymmetric kinetic model is possible. The *K*_*D*_^Na^ (2.7 ± 0.6 mm) and p*K_a_* (8.9 ± 0.1) values determined for V254C NhaA by the asymmetric kinetic model are almost identical to the values determined for WT NhaA (Table 2).

Additional fit parameters determined by the asymmetric kinetic model are the ratios of the rate constants *k*_2_^−^/*k*_1_^+^ = 7.0 ± 3.5 and *k*_1_^+^/*k*_1_^−^ = 3.4 ± 1.1. A more graphic representation of the substrate translocation steps comes from Gibbs free energies that can be calculated from the ratios of rate constants. In the case of a fully symmetric transporter like WT NhaA, the Gibbs free energy of the inside open equals that of the outside opened conformation (Fig. 5*B*). In an asymmetric transporter their Gibbs free energies differ by Δ*G* = *G_i_* − *G_o_* given by Equation 1,

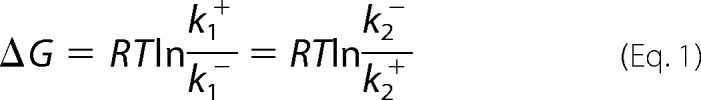
 with *R* and *T* being gas constant and temperature. According to our fit, the inside open conformation is destabilized relative to the outside open conformation by 3.4 kJ/mol.

Complementary information comes from the ratio *k*_2_^−^/*k*_1_^+^ = 7.0 ± 3.5, which allows calculation of the difference of the transition state energies ΔΔG* = ΔG*_Na_ − ΔG*_H_ of the Na^+^ (ΔG*_Na_) and H^+^ (ΔG*_H_) translocation steps as shown in Equation 2,

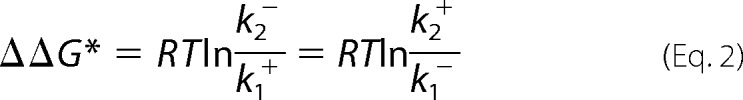


Here, the fit shows that the transition state energy of the Na^+^ translocation step is by 4.8 kJ/mol higher than that of the H^+^ translocation step, and therefore, Na^+^ translocation is rate-limiting. This is exactly the same value as for WT NhaA (9).

As residue Val-254 is part of the dimer interface (28), the asymmetric transport properties of the mutant could be the result of an altered interaction of the monomers. However, we found that the kinetic properties and pH profile of mutant ΔP45-N58 NhaA in both transport directions were virtually identical to the WT (Fig. 2, *D* and *E*). Hence, the asymmetric transport properties of V254C are probably not the result of a changed interaction of the monomers.

As mentioned above, we can alternatively account for the experimentally observed asymmetry by choosing asymmetric properties of the substrate-binding site, and indeed this model gives equally good fits to the data. Why then is the kinetic model outlined above preferable? First, a mutation located at 20 Å distance from the substrate-binding site is more plausible to affect the conformation of the protein than the binding site. Second, the model yields sensible dynamic parameters, like a transition state energy difference for the substrate translocation steps that is identical to that of the WT. In addition, the analysis shows that altered pH profiles are not necessarily related to changing substrate binding properties but that changing the conformational states of the protein can have similar results.

How could the replacement of Val-245 by a cysteine alter the conformational equilibrium of the transporter? Val-254 is located on helix IX at the border between the membrane and the cytoplasm (6, 16, 25, 28). It has also been shown that this part of helix IX and loop VIII/IX is involved in H^+^- and Na^+^-dependent conformational changes (7, 9, 13, 29, 30). Because of the fact that position 254 is located at the border between membrane and cytoplasm, it probably is in closer contact with the apolar membrane environment in the inside open conformation (like in the crystal structure, Fig. 1) than in the inside closed or outside open conformation. Replacement of the apolar valine 254 by a polar cysteine could therefore destabilize the inward-facing conformation as proposed from our kinetic analysis.

##### Low Affinity Substrate Binding in H225R NhaA

In agreement with the literature (12), the pH dependence of H225R NhaA is shifted by approximately 1 pH unit to the acidic compared with the wild type (Fig. 2*A*). Using a simultaneous fit of the pH and sodium dependences to the symmetric kinetic model (Fig. 4, *E* and *F*), it becomes apparent that the substrate-binding site of the H225R mutant shows more than 20-fold reduced affinity for both substrates Na^+^ and H^+^ (Table 2). In comparison with the WT, the p*K_a_* of the mutant p*K_a_* = 7.1 is shifted by ∼1.7 pH units to the acidic range (Table 2), and the dissociation constant for Na^+^ is *K*_*D*_^Na^ ≈86 mm compared with *K*_*D*_^Na^ ≈3 mm in the WT.

When changing from pH 8 to pH 7, the *K*_*m*_^Na^ value of H225R NhaA increased by only 50%. Hence, the increase of the *K*_*m*_^Na^ values after lowering the pH to suboptimal values is smaller for H225R NhaA than for WT NhaA (Table 1). However, for both WT and mutant, the decrease of the transporter activity at low pH values can be fully accounted for by an increase of the *K*_*m*_^Na^ values. No change in *V*_max_ is observed as demonstrated by the good quality of the simultaneous fit, which implicitly uses a pH-independent *V*_max_. In consequence, acidic down-regulation in this low affinity mutant functions according to the same competitive mechanism as proposed for WT NhaA.

The mutation is located in the periplasmic ion passage at a distance of ∼15 Å from the substrate-binding site (6, 15). In the reverse transport mode, the introduction of the positively charge arginine could hinder Na^+^ passing from the periplasmic medium to the substrate-binding site and explain the increased *K*_*D*_^Na^. However, it does not explain the p*K* shift, because H^+^ binds from the cytoplasmic side. Therefore, a direct interaction of the introduced positive charge with the binding site via electrostatic networks is more likely as suggested before (31, 32). A more concrete structure-based explanation for the changed substrate binding affinities is lacking as the structure of NhaA in the outside open conformation is not available.

##### Slow H^+^ Translocation in A167P NhaA?

An increase in the transport rate of a secondary transporter by an addition of a counter ion (K^+^ in the presence of valinomycin) that collapses the potential gradient has often been taken as a canonical criterion for a transporter with an electrogenic stoichiometry without directly measuring it (3). As shown here, in marked contrast to the WT NhaA, A167P NhaA shows an apparent indifference to membrane potential; its ΔpH-driven Na^+^ uptake rate into proteoliposomes is not accelerated by collapsing of the membrane potential (Fig. 3*A*). Therefore, it was very surprising to find out by SSM-based electrophysiology that ion transport by A167P NhaA is clearly electrogenic (Fig. 3*B*). How can the two apparently contradictory results be reconciliated?

Based on our electrophysiological analysis (9) we have recently suggested a mechanistic model for the electrogenic behavior of WT NhaA taking into account that two negatively charged residues, Asp-163 and Asp-164, constitute the Na^+^-binding site (6, 11). Therefore, the conformational transition of the Na^+^-loaded carrier (C_i_Na → C_o_Na) is associated with the displacement of two negatively charged aspartate residues plus the Na^+^ ion resulting in the displacement of one net negative charge. Indeed, Na^+^ translocation was experimentally verified to generate a negative charge displacement (9). In contrast, during the conformational transition of the H^+^-loaded carrier (C_o_H → C_i_H), the two H^+^ ions fully compensate the two negative aspartate charges leading to an electroneutral reaction. Because in WT NhaA the electrogenic Na^+^ translocation is rate-limiting (9), turnover is strongly voltage-dependent.

Coming back to A167P NhaA, we note that it has a strongly reduced *V*_max_ (Table 1) and sodium uptake capacity (Fig. 3*A*). Now, we propose that the A167P mutation selectively slows down H^+^ translocation to an extent that this step becomes rate-limiting, rendering the carrier voltage-independent as experimentally observed. Moreover, A167P NhaA shows a stronger down-regulation in the alkaline range compared with WT NhaA (Fig. 3*C*) implying rate limitation by the protonation-induced partial reaction. Additional support for this interpretation comes from kinetic analysis. The sodium dependence at pH 7.5 and 8.5 as well as the pH dependence at 10 and 100 mm Na^+^ can be simultaneously fitted with a *k*_2_/*k*_1_ ratio of 0.1 (Fig. 4, *G* and *H*), *i.e.* Na^+^ translocation in the mutant is much faster than H^+^ translocation. Unfortunately, the fit is not very sensitive to the *k*_2_/*k*_1_ ratio, and reasonable fits may also be obtained using a ratio of >1 or, in other words, with a rate-limiting Na^+^ translocation step. In this case, the apparent potential indifference of Na^+^ uptake is due to the reduced turnover of A167P NhaA, resulting in a lower membrane potential. In any case, our electrophysiological study reveals that a reduced turnover and/or a switch of the rate-limiting step from an electrogenic to an electroneutral reaction in the reaction cycle of an electrogenic secondary transporter can yield a phenotype of an electroneutral transporter.

## CONCLUSIONS

Many single site mutations of the NhaA Na^+^/H^+^ exchanger with a modified pH response have been described so far. It has always been enigmatic that these mutations are located in very different parts of the protein and can be as far as 20 Å from the substrate-binding site. In this study, we compare four mutations located in four different central helices at distances ranging from 7 to 20 Å from the binding site (Fig. 1 and Table 2) using electrophysiology and a detailed kinetic analysis. A common pattern observed in the variants is that acidic pH increases *K*_*m*_^Na^ and does not affect *V*_max_. This together with the fact that all substrate dependences can be fitted with the kinetic model of Fig. 1*B* is clear evidence for a competitive mechanism contributing to pH regulation in NhaA.

This study shows that three of the four mutations affect very different aspects of the NhaA transport mechanism (Table 2). We find mutation with impaired Na^+^ (A167P) or H^+^ binding (G338S) or both (H225R). But also the conformational dynamics of the protein may be altered by the mutation. In A167P NhaA, turnover is slowed down and possibly H^+^ translocation becomes rate-limiting, which explains its apparent voltage insensitivity. For the asymmetric mutant V254C NhaA, it is proposed that the inside open conformation is destabilized relative to the outside open conformation. Our comparison shows that the specific effect of a mutation does not correlate with its distance to the binding site. Possible mechanisms are long range electrostatic networks (31, 32), overall structural perturbations, or a modification of the ion access pathways. To solve this problem, a higher resolution structure, including the bound cation, and a structure of the outside open conformation of NhaA are badly needed.

## Footnotes

3 D. Alkoby and E. Padan, unpublished results.

## Footnotes

2 The abbreviations used are:

SSM: solid supported membrane
ISO: inside-out
RSO: right-side out.
